# Trajectory of parental health-related quality of life after neonatal hospitalization – a prospective cohort study

**DOI:** 10.1186/s12955-025-02345-3

**Published:** 2025-03-15

**Authors:** Julia Karsch, Mascha Schönfeld, Ann-Kathrin Mühler, Susanne Tippmann, Christine Arnold, Michael S. Urschitz, Eva Mildenberger, André Kidszun

**Affiliations:** 1 Division of Neonatology, Center for Pediatric and Adolescent Medicine, University Medical Center of the Johannes Gutenberg-University Mainz, Mainz, Germany; 2 Division of Neonatology, Department of Pediatrics, Inselspital, Bern University Hospital, University of Bern, Bern, Switzerland; 3 Division of Pediatric Epidemiology, Institute for Medical Biostatistics, Epidemiology and Informatics, University Medical Center of the Johannes Gutenberg-University Mainz, Mainz, Germany

**Keywords:** NICU, Prospective cohort study, Parents, Postpartum, Health-related quality of life

## Abstract

**Background:**

Health-related quality of life is an important measure of patient-reported outcomes. There is limited evidence on how parental health-related quality of life develops after neonatal hospitalization.

**Objective:**

To evaluate parents’ health-related quality of life (HRQL) during the year following their infant’s treatment in the neonatal intensive care unit (NICU).

**Methods:**

This prospective cohort study, conducted at a German university hospital between 2020 – 2023, examined HRQL among parents of infants hospitalized in the NICU for ≥ 14 days and parents of infants discharged from the maternity ward according to PedsQL™ Family Impact Module. Multiple linear regression analysis was performed to identify associations between cohort affiliation and differences in parental HRQL. Key secondary outcome was parenting sense of competence (PSOC).

**Results:**

Participants included 131 parents of NICU infants and 122 unexposed parents. HRQL increased over time for NICU mothers (58.7 at 14 days, 70.8 at 6 months, 77.0 at 12 months after birth) and NICU fathers (69.8 at 14 days, 73.9 at 6 months, 75.7 at 12 months). NICU treatment was significantly associated with lower HRQL at 14 days (mothers: -20.26 points; *P* < .001; fathers: -9.40 points; *P* = .04), but not at 6 or 12 months after birth. At 12 months postpartum, NICU mothers showed higher PSOC compared to unexposed mothers (mean difference -4.85; *P* = 0.005).

**Conclusions:**

Parents and especially mothers of NICU infants reported lower HRQL at 14 days postpartum. At 6 and 12 months, their HRQL improved, aligning with that of unexposed parents.

## Introduction

Having a baby who requires medical care immediately after birth presents a unique set of stressors and challenges for parents [1, 2]. Parents may experience uncertainty regarding their infant's health [3] and struggle with self-blame for their baby's preterm birth or cause of hospitalization [4]. Dependence on medical staff for instruction and mediation of caregiving activities can initially complicate parent-infant attachment and create feelings of parental inadequacy [5]. After discharge, parents assume primary responsibility for their infant’s potentially extensive ongoing medical care [6, 7]. These experiences can have serious implications for parents’ mental health [8–10], which may in turn affect parent-infant interactions [11] and adversely affect the infant’s development [12, 13].

Health-related quality of life (HRQL) describes “[…] those aspects of self-perceived well-being that are […] affected by the presence of disease or treatment” [14]. Parental HRQL holds intrinsic value as an indicator of parental well-being, as influenced by the medical treatment of their infant, while also potentially having implications for the long-term development of the infant [13]. Both, neonatologists and parents of NICU patients, consider HRQL to be a significant factor in the decision-making process regarding treatment options [15]. In addition, parents of NICU patients consider HRQL, family functioning, and parental well-being to be important elements of meaningful outcomes in neonatology [16].

There is a paucity of research examining the longitudinal assessment of HRQL among parents of term and preterm infants in the NICU, as well as the HRQL of mothers compared to fathers.[17, 18]. The objective of this study was to assess the gender-specific HRQL of parents during the first year following their infant's treatment in the NICU in comparison to parents of infants discharged from the maternity ward. We hypothesized that NICU parents would report lower HRQL compared to unexposed parents.

## Methods

This study was approved by the responsible Ethics Committee of the Medical Association of Rhineland-Palatinate. All participants provided written informed consent before participation. This report follows the Strengthening the Reporting of Observational Studies in Epidemiology (STROBE) reporting guidelines.

### Study design and setting

This prospective clinical cohort study was conducted at a German university hospital between 2020 and 2023. Approximately 2000 infants are delivered at this medical center on an annual basis. The center’s tertiary care NICU admits infants of all gestational ages (GA), with up to 75 preterm infants with a birth weight ≤ 1500 g admitted per year. While most of its patients are inborn, this NICU also accepts transfers from hospitals with lower levels of care. Although rooming-in for parents is not routinely available, parents are encouraged to participate in their infants' care to the greatest extent possible and are permitted to visit the unit at any time. Subject to availability, parents are offered free accommodation in an adjacent Ronald McDonald House. Given that a significant portion of the study was conducted during the ongoing pandemic, visitor restrictions permitted only one parent to visit at a time.

### Participants and procedure

Parents of infants treated in the NICU for ≥ 14 days and parents of infants discharged home from the maternity ward were eligible for inclusion. Parents were excluded if they had a psychiatric illness requiring medical treatment (self-report) or if their infant was outborn or died within the first 14 days of life. Non-German speaking families were also excluded.

Eligible parents were contacted by research assistants or pediatric residents. Participant recruitment took place from January 2020 to January 2022. During the first four months of recruitment, physical copies of the surveys were mailed to parents who agreed to participate. Due to COVID-19-related visitation restrictions and to facilitate participation, questionnaires were later digitalized and provided online.

Participants were instructed to complete questionnaires at 14 days, 6 months, and 12 months postpartum. Questionnaires included the Pediatric Quality of Life Family Impact Module (PedsQL™ FIM) [19], demographic questions, and the Parenting Sense of Competence Scale (PSOC) [20, 21] at 6 and 12 months postpartum. Participants who did not complete the 6-month questionnaire but did complete the 12-month questionnaire were not excluded from analysis.

### Study outcomes

Primary outcome of the study was parental HRQL as measured by the PedsQL™ FIM sum score at 6 months postpartum. The PedsQL™ FIM is a 36-item instrument designed to measure the impact of pediatric chronic health conditions on parents and family [19]. It consists of eight subdomains (physical, emotional, social, and cognitive functioning; communication; worry; family relationships; and daily activities). Parents were asked to rate the frequency at which they felt statements such as "I felt tired throughout the day" applied to them on a five-point Likert scale. Items are reverse scored and transformed to a scale of 0–100, with higher scores indicating higher HRQL. If more than 50% of the items in a participant’s questionnaire were missing, their response was excluded from analysis.

Secondary outcomes were parental HRQL at 14 days and 12 months postpartum and self-rated parental competence at 6 and 12 months postpartum. Parental competence was measured using the German version of the PSOC, which consists of 16 items [21]. Parents were asked to rate their level of agreement with each item on a 6-point Likert scale. Higher scores indicate a greater sense of competence, “Efficacy”, or “Satisfaction”, with scores for “Efficacy” and “Satisfaction” subscales ranging from 7 to 42 and 9 to 54, and total scores ranging from 16 to 96.

### Statistical analysis

Participants were characterized using descriptive statistics. Mothers and fathers from each cohort were analyzed independently. Effect sizes for between-cohort differences in outcomes were calculated using Cohen's *d*.

Within cohorts, changes in parental HRQL were assessed using one-way repeated measures ANOVA and post hoc tests. Only parents who completed both follow-up surveys were included in these analyses. Greenhouse–Geisser adjustments were employed to correct for violations of sphericity among mothers in the NICU group and fathers in the reference group.

Multiple linear regression analysis was performed with cohort affiliation as primary explanatory variable and HRQL as the dependent variable. The effect estimate B and its 95% confidence interval were adjusted for potential confounders (infant GA, parental age, relationship status, previous children, education, twin status, and level of employment before the current pregnancy). In cases where participants provided missing values for an independent categorical variable, dichotomous dummy variables were created for each missing response option. Missing values for metric scaled variables were imputed by using the mean of participants of the same sex for the respective variable. Some follow-up questionnaires (4 within the NICU cohort, 6 within the unexposed cohort) featured pseudonymized IDs with transposed digits, which prevented a clear assignment to the corresponding IDs at first participation, 14 days postpartum. These participants were therefore excluded from the 6- and 12-month regression analyses.

Between-cohort differences in parenting competence were analyzed using independent t-tests. Due to the exploratory nature of the study, no a priori sample size calculation was performed, and recruitment ended in January 2022 due to lack of human resources. Statistical analyses were performed using IBM® SPSS® Statistics 23.

## Results

A total of 253 parents from 161 families were enrolled at baseline (14 days postpartum), 131 parents in the NICU group (58 fathers, 73 mothers) and 122 unexposed parents (39 fathers, 83 mothers). Parent and infant characteristics are summarized in Tables 1 and 2.

**Table 1:** Parent characteristics

	**NICU** **Mothers** (*n* = 73)	**NICU** **fathers** (*n* = 58)	**Unexposed mothers** (*n* = 83)	**Unexposed** **Fathers** (*n* = 39)
	**families** (*n* = 77)		**families** (*n* = 84)	
**Relationship status,** n (%)				
Single	0 (0)		3 (4)	
Living together	13 (17)		17 (20)	
Married	62 (80)		62 (74)	
Missing “relationship status”	2 (3)		2 (2)	
**Number of older children,** n (%)				
0	53 (69)		40 (48)	
1	18 (23)		34 (40)	
2	6 (8)		8 (10)	
3 or more	0 (0)		2 (2)	
**Singleton pregnancy**, n (%)	60 (78)		82 (98)	
**Age**, Mean (SD), years	33.2 (4.1)	35.0 (5.1)	33.1 (4.4)	34.9 (5.6)
**Education,** n (%)				
Did not graduate	1 (1)	1 (2)	0 (0)	0 (0)
Graduated secondary school	9 (12)	11 (19)	9 (10)	1 (2)
Higher education entrance level qualification *(Abitur)*	12 (17)	8 (13)	16 (19)	7 (18)
University degree	51 (70)	37 (64)	55 (68)	31 (80)
Missing “education”	0 (0)	1 (2)	3 (3)	0 (0)
**Occupation before current pregnancy,** n (%)				
Not working	1 (1)	1 (2)	2 (2)	0 (0)
Apprenticeship	2 (3)	0 (0)	0 (0)	0 (0)
Studying	3 (4)	2 (3)	3 (4)	4 (10)
Part-time < 50%	4 (6)	1 (2)	11 (14)	1 (3)
Part-time > 50% or full-time	63 (86)	53 (91)	65 (78)	34 (87)
Missing “occupation before current pregnancy”	0 (0)	1 (2)	2 (2)	0 (0)
**Monthly net income**, n(%)				
≤ 2000 €	12 (16)	3 (5)	16 (19)	3 (8)
> 2000 €	53 (73)	46 (79)	57 (69)	25 (64)
≥ 5000 €	8 (11)	7 (12)	7 (8)	11 (28)
Missing “monthly net income”	0 (0)	2 (4)	3 (4)	0 (0)
**Planned parental leave,** mean (SD), months	16.26 (7.4)	3.28 (3.6)	17.48 (8.8)	2.82 (2.5)
Missing “planned parental leave”, n (%)	1 (1)	1 (2)	0 (0)	0 (0)
**Planned resumption of professional activity after ongoing pregnancy**, n(%)				
To full extent	10 (14)	48 (83)	21 (25)	34 (87)
To reduced extent	50 (69)	4 (7)	57 (69)	2 (5)
Not sure yet	9 (12)	4 (7)	4 (5)	2 (5)
Family work longterm	4 (5)	0 (0)	1 (1)	0 (0)
Missing “planned resumption of professional activity after ongoing pregnancy”	0 (0)	2 (3)	0 (0)	1 (3)

*NICU* = Neonatal intensive care unit, *SD* = Standard deviation

**Table 2:** Infant characteristics

	**NICU cohort (*n* = 94)**	**Unexposed cohort (*n* = 86)**
**Gestational age of the child,** mean (SD), weeks	31.6 (4.3)	39.2 (1.4)
Missing “gestational age”, n (%)	0 (0)	1 (1)
**Weight of child,** mean (SD), g	1809.6 (877.5)	3498.1 (508.6)
Missing “weight of child”, n (%)	4 (4)	0 (0)
**Male**, n (%)	54 (57)	48 (56)
**Female**, n (%)	36 (38)	38 (44)

*NICU* = Neonatal intensive care unit, *SD* = Standard deviation

**Fig. 1:**
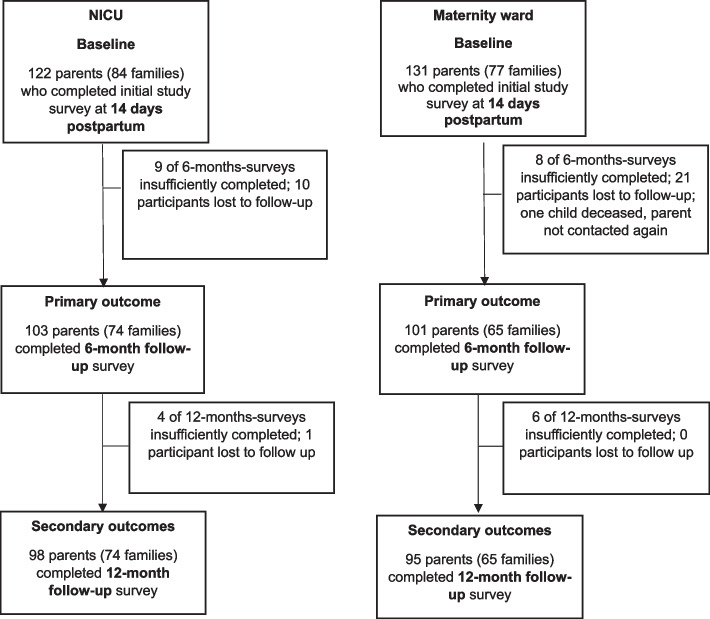
Study flow-diagram. Lines and arrows indicate participant flow throughout the study. NICU = Neonatal Intensive Care Unit

Figure 1 summarizes participant flow throughout the study. There were no significant differences between parents who completed all questionnaires and those who were lost to follow-up.

### Parental health-related quality of life

**Fig. 2:**
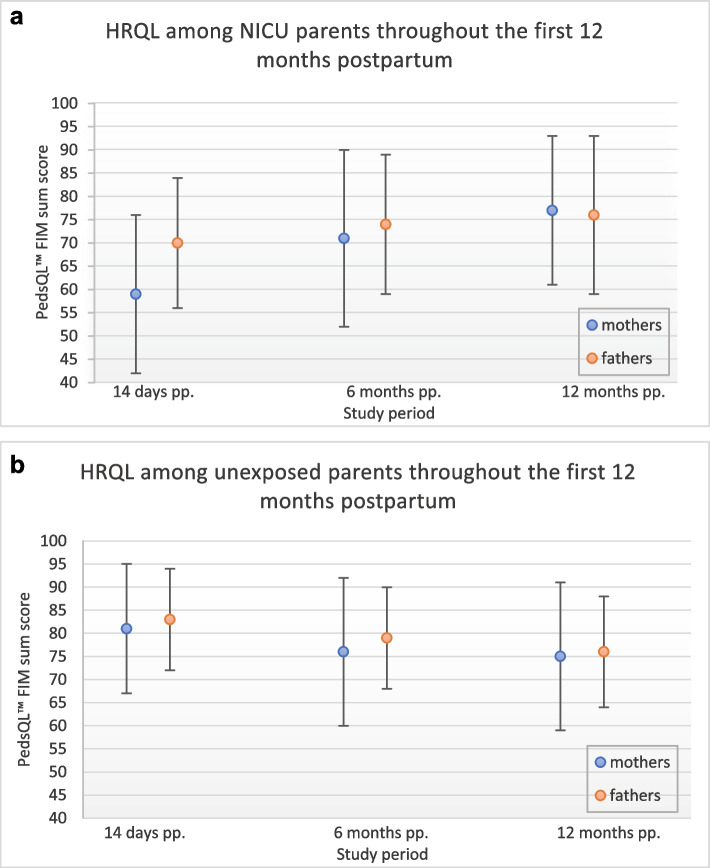
**a** Mean PedsQL™ FIM sum scores in the NICU cohort throughout the course of the study. PedsQL™ FIM scores range from 0–100 with higher scores indicating increased HRQL. Circles indicate mean PedsQL™ FIM sum scores, error bars indicate standard deviation. PedsQL™ FIM = Pediatric Quality of Life Inventory Family Impact Module, NICU = Neonatal Intensive Care Unit, pp. = postpartum **b** Mean PedsQL™ FIM sum scores in the unexposed cohort throughout the course of the study. PedsQL™ FIM scores range from 0–100 with higher scores indicating increased HRQL. Circles indicate mean PedsQL™ FIM sum scores, error bars indicate standard deviation. PedsQL™ FIM = Pediatric Quality of Life Inventory Family Impact Module, pp. = postpartum

HRQL increased significantly from 14 days to 6 months postpartum for mothers in the NICU group (mean difference + 11.73; *P* < 0.001), while decreasing over the same period for unexposed mothers (mean difference −4.30; *P* = 0.03). HRQL among fathers in the NICU group remained relatively stable over the course of the study, whereas HRQL among unexposed fathers decreased significantly from 14 days to 12 months postpartum (mean difference −7.09; *P* = 0.01) (Table 3; Fig. 2a and b).

**Table 3:** Within- subgroup HRQL-changes among mothers and fathers in each cohort

	**Mean differences in PedsQL™ FIM sum scores among NICU cohort mothers** (*n* = 50)			**95% CI**	
		**SE**	***P*** **value**	**LCI**	**UCI**
T1 to T2	11.73^*^	1.94	< .001	6.91	16.55
T1 to T3	15.31^*^	2.53	< .001	9.03	21.58
T2 to T3	3.57	1.86	.18	−1.04	8.19
	**Mean differences in PedsQL™ FIM sum scores among unexposed cohort mothers** (*n* = 59)			**95% CI**	
		**SE**	***P*** **value**	**LCI**	**UCI**
T1 to T2	−4.30^*^	1.58	.03	−8.22	−0.39
T1 to T3	−4.74^*^	1.64	.02	−8.80	−0.69
T2 to T3	−0.44	1.29	> .99	−3.62	2.75
	**Mean differences in PedsQL™ FIM sum scores among NICU cohort fathers** (*n* = 32)			**95% CI**	
		**SE**	***P*** **value**	**LCI**	**UCI**
T1 to T2	3.02	2.47	.69	−3.23	9.27
T1 to T3	4.17	3.10	.56	−3.67	12.00
T2 to T3	1.15	2.49	> .99	−5.15	7.45
	**Mean differences in PedsQL™ FIM sum scores among unexposed cohort fathers** (*n* = 26)			**95% CI**	
		**SE**	***P*** **value**	**LCI**	**UCI**
T1 to T2	−4.72	2.08	.10	−10.05	0.62
T1 to T3	−7.09^*^	2.16	.01	−12.64	−1.54
T2 to T3	−2.37	1.14	.15	−5.31	0.56

One-way repeated-measures ANOVA post-hoc tests to evaluate within-subgroup HRQL-changes among parents who completed all follow-up surveys throughout the course of the study

T1 = 14 days postpartum, T2 = 6 months postpartum, T3 = 12 months postpartum. NICU = neonatal intensive care unit, PedsQL™ FIM = Family Impact Module, *SE =* standard error, *LCI* = lower confidence interval, *UCI* = upper confidence interval, *CI* = confidence interval

The convergence of overall HRQL scores in both cohorts appeared to result from relatively evenly distributed increases in the NICU group and decreases in the unexposed group across all subdomain scores. Table 4 shows complete PedsQL™ FIM scores for mothers in both cohorts throughout the study. Results for fathers from both cohorts are shown in Table 5.

**Table 4:** Overview of PedsQL™ FIM subdomain scores for mothers in both cohorts throughout the study

	**NICU cohort** **14 days** (*n* = 73)	**Unexposed cohort** **14 days** **(***n* = 83)	**Cohen’s** ***d***	**NICU cohort** **6 months** **(***n* = 61)	**Unexposed cohort** **6 months** **(***n* = 69)	**Cohen’s** ***d***	**NICU cohort** **12 months** (*n* = 58)	**Unexposed cohort** **12 months** (*n* = 68)	**Cohen’s** ***d***
**PedsQL FIM sum score**, mean (SD)	58.7 (16.9)	81.4 (13.6)	−1.5	70.8 (19.0)	76.0 (15.9)	- .30	77.0 (15.8)	75.1 (15.6)	.11
**Physical functioning,** mean (SD)	49.7 (21.8)	71.2 (19.0)	−1.06	65.8 (23.6)	67.6 (19.8)	- .08	69.1 (20.0)	65.7 (21.6)	.12
**Emotional functioning**, mean (SD)	54.1 (21.8)	83.6 (16.7)	−1.53	71.9 (26.3)	78.2 (18.3)	- .28	80.5 (18.1)	75.0 (18.7)	.32
**Social functioning**, mean (SD)	55.7 (23.3)	81.3 (19.3)	−1.20	69.0 (24.8)	75.8 (22.4)	- .29	77.9 (20.9)	74.9 (21.7)	.14
**Cognitive functioning**, mean (SD)	59.4 (29.4)	81.2 (20.1)	- .88	72.2 (24.6)	74.1 (23.1)	- .08	77.4 (20.4)	72.4 (23.7)	.24
**Communication**, mean (SD)	68.5 (22.5)	92.5 (11.5)	−1.37	77.5 (22.6)	87.9 (15.6)	- .54	82.3 (20.3)	86.5 (17.6)	- .22
**Worry**, mean (SD)	68.8 (21.9)	93.4 (11.7)	−1.43	75.6 (21.3)	87.0 (18.6)	- .57	82.6 (20.0)	90.2 (13.3)	- .46
Missing Worry, n (%)	1 (1)	1 (1)		1 (2)	3 (4)		0 (0)	2 (3)	
**Daily activities**, mean (SD)	31.7 (24.9)	61.9 (29.9)	−1.1	56.3 (28.0)	62.8 (23.8)	- .25	69.7 (24.4)	65.8 (26.2)	.15
Missing Daily Activities, n (%)	0 (0)	2 (2)		0 (0)	2 (3)		0 (0)	2 (3)	
**Family relationships**, mean (SD)	76,9 (20.6)	85.3 (19.0)	- .42	75.9 (21.0)	76.1 (21.5)	- .01	76.8 (21.1)	73.4 (23.0)	.16
Missing Family Relationships**,** n (%)	0 (0)	2 (2)		0 (0)	2 (3)		0 (0)	1 (1)	

PedsQL™ FIM scores range from 0–100 with higher scores indicating increased HRQL. Cohen's *d* illustrates effect sizes of differences in results among mothers of both cohorts

*NICU* = Neonatal intensive care unit, *FIM* = Family Impact Module, *HRQL* = Health-related quality of life, *SD* = Standard deviation

**Table 5:** Overview of PedsQL™ FIM subdomain scores for fathers in both cohorts throughout the study

	**NICU** **cohort** **14 days** (*n* = 58)	**Unexposed** **cohort** **14 days** (*n* = 39)	**Cohen’s** ***d***	**NICU** **cohort** **6 months** (*n* = 40)	**Unexposed cohort 6 months** (*n* = 34)	**Cohen’s** ***d***	**NICU cohort** **12 months** (*n* = 37)	**Unexposed cohort** **12 months** (*n* = 30)	**Cohen’s** ***d***
**PedsQL FIM sum score**, mean (SD)	69.8 (13.9)	83.1 (10.6)	−1.05	73.9 (14.6)	79.0 (11.0)	- .38	75.7 (17.3)	76.1 (11.8)	- .03
**Physical Functioning**, mean (SD)	64.3 (21.0)	75.3 (16.8)	- .56	67.4 (21.6)	71.1 (14.4)	- .20	70.4 (19.8)	65.8 (16.7)	.25
**Emotional Functioning** mean (SD)	69.2 (19.7)	88.4 (13.9)	−1.08	80.9 (18.3)	86.0 (10.1)	- .34	81.8 (17.9)	83.2 (15.4)	- .08
**Social Functioning**, mean (SD)	67.7 (20.7)	78.4 (18.4)	- .54	70.1 (21.7)	77.2 (17.3)	- .36	72.6 (24.3)	71.3 (19.0)	.06
**Cognitive Functioning**, mean (SD)	76.0 (18.8)	82.6 (15.6)	- .37	70.6 (22.7)	77.9 (15.2)	- .37	72.3 (23.6)	76.8 (16.7)	- .22
**Communication**, mean (SD)	79.0 (19.6)	93.8 (11.7)	- .88	82.2 (16.2)	93.1 (9.1)	- .82	83.8 (19.0)	91.4 (9.7)	- .49
**Worry**, mean (SD)	70.6 (20.3)	93.5 (10.9)	−1.33	83.1 (15.5)	87.9 (14.8)	- .32	84.3 (20.0)	90.3 (12.5)	- .35
Missing Worry, n (%)	0 (0)	0 (0)		1 (2)	0 (0)		2 (5)	0 (0)	
**Daily Activities**, mean (SD)	46.3 (25.6)	63.7 (23.3)	- .70	59.2 (28.5)	63.5 (21.8)	- .17	66.0 (28.7)	59.4 (26.1)	.24
Missing Daily Activities, n (%)	1 (2)	0 (0)		0 (0)	0 (0)		0 (0)	0 (0)	
**Family Relationships**, mean (SD)	80.1 (17.6)	85.9 (14.3)	- .35	75.9 (17.4)	77.5 (15)	- .10	75.1 (23.4)	71.2 (17.2)	.19
Missing Family Relationships, n (%)	1 (2)	0 (0)		0 (0)	0 (0)		0 (0)	0 (0)	

PedsQL™ FIM scores range from 0 – 100 with higher scores indicating increased HRQL. Cohen's *d* illustrates effect sizes of differences in results among fathers of both cohorts

PedsQL™ FIM = Pediatric Quality of Life Inventory Family Impact Module, NICU = Neonatal intensive care unit, HRQL = Health-related quality of life, SD = Standard deviation

Adjusted regression models of the associations of NICU treatment with HRQL are shown as Tables 6 and 7.

**Table 6:** Adjusted associations between cohort affiliation and HRQL, maternal participants

						**95% CI**	
	**B**	**SE**	**β**	**T**	***P* value**	**LCI**	**UCI**
NICU vs. unexposed T1 (*n* = 156)	−20.26	4.14	- .54	−4.89	< .001	−28.44	−12.08
NICU vs. Unexposed T2 (*n* = 125)	−4.77	5.18	- .14	- .92	.36	−15.05	5.50
NICU vs. Unexposed T3 (*n* = 122)	−1.18	4.43	- .04	- .27	.79	−9.97	7.59

Multiple linear regression analysis with health-related quality of life (measured using PedsQL™ FIM sum score) as dependent variable, maternal participants 14 days (T1), 6 months (T2) and 12 months (T3) postpartum. All multiple linear regression models were adjusted for potential confounders (infant’s gestational age, parental age, relationship status, previous children, education, twin status and extent of professional occupation before current pregnancy)

HRQL: Health-related quality of life, PedsQL™ FIM = Pediatric Quality of Life Inventory Family Impact Module, B = adjusted mean difference, SE = Standard error, β = Standardized mean difference, LCI = Lower confidence interval, UCI = Upper confidence interval, CI = Confidence interval, R = Multiple correlation coefficient, R^2^ = Multiple coefficient of determination

T1.: *n* = 156; R^2^ = .42; corrected R^2^ = .34; F (20.40) = 4.95; *P* < .001

T2.: *n* = 125; R^2^ = .10; corrected R^2^ =—.06; F (19.11) = .62; *P* = .88

T3.: *n* = 122; R^2^ = .17; corrected R^2^ = .03; F (18.10) = 1.19; *P* = .28

**Table 7:** Adjusted association between cohort affiliation and HRQL, paternal participants

						**95% CI**	
	**B**	**SE**	**β**	**T**	***P* value**	**LCI**	**UCI**
NICU- vs. unexposed T1 (*n* = 97)	−9.40	4.39	- .33	−2.14	.04	−18.14	- .65
NICU vs. unexposed T2 (*n* = 71)	−4.34	4.76	- .17	- .91	.37	−13.89	5.22
NICU vs. unexposed T3 (*n* = 64)	−1.79	6.49	- .060	- .28	.78	−14.87	11.28

Multiple linear regression analysis with health-related quality of life (measured using PedsQL™ FIM sum score) as dependent variable, paternal participants, 14 days (T1), 6 months (T2) and 12 months postpartum (T3)

All multiple linear regression models were adjusted for potential confounders (infant’s gestational age, parental age, relationship status, previous children, education, twin status and extent of professional occupation before current pregnancy)

HRQL = Health-related quality of life, PedsQL™ FIM = Pediatric Quality of Life Inventory Family Impact Module, B = adjusted mean difference, SE = Standard error, β = Standardized mean difference, LCI = Lower confidence interval, UCI = Upper confidence interval, CI = Confidence interval, R = Multiple correlation coefficient, R^2^ = Multiple coefficient of determination

T1: *n* = 97; R^2^ = .37; corrected R^2^ = .23; F (18.78) = 2.56; *P* = .002

T2: *n* = 71; R^2^ = .40; corrected R^2^ = .20; F (17.53) = 2.04; *P* = .03

T3: *n* = 64; R^2^ = .25; corrected R^2^ =—.01; F (16.47) = .97; *P* = .50

After adjusting for confounders, NICU treatment was significantly associated with lower HRQL among parents at 14 days (mothers: B = −20.26; 95% confidence interval (CI): −28.44 to −12.08; *P* < 0.001; fathers: B = −9.40; 95% CI: −18.14 to −0.65; *P* = 0.04), but not at 6 (mothers: B = −4.77, 95% CI: −15.05 to 5.50; *P* = 0.36; fathers: B = −4.34; 95% CI: −13.89 to 5.22; *P* = 0.37) or 12 months (mothers: B = −1.18; 95% CI: −9.97 to 7.59; *P* = 0.79; fathers: B = −1.79; 95% CI −14.87 to 11.28; *P* = 0.78).

### Self-rated parental competence

PSOC scores for mothers in both cohorts are shown in Table 8. Mothers in the NICU group had similar total PSOC scores at 6 months postpartum compared to unexposed mothers. Their total PSOC scores (+ 4.8 points) and their subscores (“Satisfaction” (+ 3.9 points) and “Efficacy” (+ 0.9 points)) increased at 12 months postpartum, while PSOC scores of unexposed mothers decreased or remained essentially the same. This resulted in higher PSOC scores for mothers in the NICU group compared to unexposed mothers by the end of the study (mean difference in total PSOC scores: −4.85; 95% CI −8.2 to −1.51; *P* = 0.005). Table 9 shows PSOC results for fathers. Unexposed fathers initially showed higher parenting satisfaction than fathers in the NICU group (mean difference in satisfaction scores: 3.27; 95% CI 0.61 to 5.92; *P* = 0.02). Their PSOC scores also increased from 6 to 12 months but were not higher than those of unexposed fathers at the end of the study (mean difference in total PSOC sores: −1.84; 95% CI −6.02 to 2.33; *P* = 0.38).

**Table 8:** Self-rated parental competence among mothers in both cohorts measured using Parenting Sense of Competence Scale

	**NICU cohort** **6 months** (*n* = 61)	**Unexposed** **cohort** **6 months** (*n* = 69)	***P* value**	**Cohen’s *d***	**NICU** **cohort** **12 months** (*n* = 58)	**Unexposed cohort** **12 months** (*n* = 68)	***P* value**	**Cohen’s *d***
**PSOC total score**, mean (SD)	70 (9.1)	70.6 (9.6)	.70	- .07	74.8 (9)	69.9 (9.5)	.005	.53
Missing “Total score”, n (%)	2 (3)	4 (6)			1 (2)	1 (1)		
**PSOC Satisfaction**, mean (SD)	37.8 (6)	39.6 (6.7)	.11	- .29	41.6 (6.3)	38.8 (6.3)	.02	.44
Missing “Satisfaction”, n (%)	2 (3)	4 (6)			1 (2)	1 (1)		
**PSOC Self-efficacy**, mean (SD)	32.2 (4.4)	31 (4.3)	.14	.28	33.1 (3.8)	31.1 (4.3)	.008	.49
Missing “Self-efficacy”, n (%)	2 (3)	4 (6)			1 (2)	1 (1)		

Parenting Sense of Competence Scale was administered at 6 and 12 months postpartum respectively. Total scores range from 16 – 96. Higher scores indicate increased parenting sense of competence. Cohen's *d* illustrates effect sizes in differences in results between NICU mothers and unexposed mothers and NICU fathers and unexposed fathers. NICU = Neonatal intensive care unit, PSOC = Parenting sense of competence scale, SD = Standard deviation

**Table 9:** Self-rated parental competence among fathers in both cohorts measured using Parenting Sense of Competence Scale

	**NICU cohort** **6 months** (*n* = 40)	**Unexposed** **cohort** **6 months** (*n* = 34)	***P*** **value**	**Cohen’s** ***d***	**NICU** **cohort** **12 months** (*n* = 37)	**Unexposed** **cohort** **12 months** (*n* = 30)	***P*** **value**	**Cohen’s** ***d***
**PSOC Total score,** Mean (SD)	69.4 (8.7)	72.8 (9)	.13	- .39	73.5 (8.8)	71.7 (7.8)	.38	.22
Missing “Total Score”, n (%)	1 (3)	0 (0)			0 (0)	1 (3)		
**PSOC Satisfaction,** Mean (SD)	38 (5.3)	41.2 (6)	.02	- .57	42 (6)	40 (5.9)	.19	.34
Missing “Satisfaction”, n (%)	1 (3)	0 (0)			0 (0)	1 (3)		
**PSOC Self-efficacy,** Mean (SD)	31.4 (4.6)	31.5 (5.3)	> .99	- .02	31.5 (4.2)	31.6 (3.6)	.91	- .03
Missing “Self-efficacy”, n (%)	1 (3)	0 (0)			0 (0)	1 (3)		

Parenting Sense of Competence Scale was administered at 6 and 12 months postpartum respectively. Total scores range from 16 – 96. Higher scores indicate increased parenting sense of competence. Cohen's *d* illustrates effect sizes in differences in results between NICU mothers and unexposed mothers and NICU fathers and unexposed fathers. NICU = Neonatal intensive care unit, PSOC = Parenting sense of competence scale, SD = Standard deviation

## Discussion

In this study, parents of neonates hospitalized in the NICU had significantly lower HRQL at 14 days postpartum compared with unexposed parents. During the first year of life, HRQL of parents of NICU infants increased steadily, while HRQL of unexposed parents decreased slightly. At 6 and 12 months after birth, parents in both cohorts had comparable HRQL scores.

Lower HRQL among parents of NICU infants at study onset may reflect differences in postpartum experiences in both cohorts. While parents of healthy infants are usually able to leave the hospital shortly after birth and establish family life at home, parents of NICU infants are often separated from their babies and initially return home without them [5]. Schuetz Haemmerli et al. suggest that parents of preterm infants have to come to terms with “the loss […] of taking for granted […] [of] handling a healthy child” [22] during the postpartum period. Accordingly, many affected parents describe struggles with loss of control [23] and parental role alteration [24, 25] as they learn to adapt to the unfamiliar environment of the NICU, where their infant is cared for by a team of medical professionals.

At 6 months postpartum, HRQL between both cohorts was no longer different. Most NICU patients had probably been discharged for several weeks at this point. Although the transition from NICU to home may be accompanied by feelings of anxiety [26], previous studies have suggested that bringing their baby home can also evoke feelings of gratitude [27] and relief [28] in parents. Several stressors commonly associated with the NICU environment are essentially eliminated as parents are no longer physically separated from their infant and the infant’s health has improved to the point where inpatient care is no longer necessary. Parents may enjoy the increased autonomy to care for their infants by themselves and to establish routines as a family outside of the NICU [26].

Participants in the unexposed cohort by contrast, have likely already lived through the challenges of parenting a newborn for several months. Transition to parenthood, even when caring for healthy infants, is associated with considerable lifestyle changes and role adjustments [29, 30], which can strain couples’ relationships [31] and, at times negatively affect their HRQL.

Studies that have previously examined parental HRQL after neonatal hospitalization report increases after discharge for most subpopulations of NICU parents that are consistent with our findings [32–35]. Our study adds to these findings by providing longitudinal normative results for HRQL of parents whose infants were not hospitalized and reinforces the understanding that this period is critical for recognizing HRQL-impairments among NICU parents. By the end of the study, mean HRQL scores were similar in both cohorts, suggesting a possible convergence of parenting experiences among study participants as hospitalization recedes into distance. The results may indicate that while parents often experience periods of emotional distress during their infant's NICU care, many can be reassured that their HRQL will "normalize" after discharge. These findings may be useful for clinicians when counseling affected parents.

Fathers are often underrepresented in studies of pediatric parent populations [36]. This study is one of the first to compare maternal and paternal HRQL trajectories from the time of hospitalization to several months after discharge. Mothers of infants in the NICU had lower HRQL compared to fathers in the same cohort. Hafeez et al. reported similar PedsQL™ FIM sum scores for mothers and fathers during the NICU stay and 3 months after discharge as in this study [34]. Previous studies have suggested that gender differences in how NICU treatment affects parents may be rooted in different parental roles and coping mechanisms during the weeks postpartum. [9, 22, 37]. Mothers often report higher levels of NICU-related stress [9, 37], anxiety, and depression [38] compared to fathers, although, as in this study, such observations do not seem to translate into significant HRQL differences between sexes 4–42 months postpartum [34, 39–41].

Mothers are inevitably more involved than fathers in some aspects of childbirth and childcare. Many preterm births are associated with significant maternal morbidity [42, 43], and mothers’ postpartum HRQL has been found to be negatively affected by maternal morbidity [44, 45] or problems in childcare tasks predominantly performed by women, such as breastfeeding difficulties [46]. In addition, mothers often appear to spend more time in the NICU, while fathers are still frequently tasked with taking on the role of “breadwinners” who are forced to divide their time between NICU and work [22, 47]. This may result in fathers experiencing less exposure to NICU-related stressors. At the same time, work also appears to represent a coping strategy for some fathers [48].

Participants' PSOC scores were similar to those of a cohort of parents of 3- to 6-year-old children surveyed within the frame of a German public health research project [21], while also showing similarities to results from the limited number of studies that have previously examined PSOC in NICU parent populations [49, 50]. Huang et al. reported slightly lower and Garfield et al. slightly higher PSOC scores for parents of preterm infants, both using the original 17-item PSOC version as opposed to the 16-item German version. Cultural aspects as well as intervention and time effects may play a role in the observed discrepancies.

Mothers of infants hospitalized in the NICU had higher PSOC scores than unexposed mothers at 12 months postpartum. Vance et al. describe that “[parenting] confidence is strengthened by effective parenting […] and the developmental success or sustained health of a child […]”[51]. NICU parents experience a somewhat delayed start to independent parenting and may not fully assume their primary caregiving role until after discharge from the hospital. It seems plausible that after several months of successful independent parenting and care of their medically fragile infants, parents' sense of parenting competence may even exceed that of parents of healthy infants.

Similar dynamics for parenting satisfaction and parenting “mastery” among mothers of very low birthweight (VLBW) and mothers of term infants as in this study were reported by Singer et al. [52]. The authors suggested that greater satisfaction and coping among mothers of VLBW infants might result from a psychological phenomenon known as “posttraumatic growth”. Herein, adverse life events induce positive personal developments in an individual’s life [53]. “Greater appreciation of life” has previously been listed as a possible outcome [54, 55]. A recent study found that up to 65% of parents of preterm infants surveyed displayed moderate to high levels of posttraumatic growth [56].

Research has also suggested that there may be reciprocal relationships between parenting self-efficacy and mental health [57] and that higher parenting self-efficacy may be associated with lower rates of maternal depression and anxiety [58]. With this in mind, it seems conceivable that increases in parental HRQL and parenting competence may be correlated within the NICU cohort in our study, and that increased parenting competence may have contributed to increases in parental HRQL, or vice versa.

### Limitations

Main limitation of the current study is its single-center design. Given the high proportion of married, university-educated participants, some degree of selection bias must be acknowledged. Non-German speaking parents and parents with psychiatric conditions requiring medical treatment were excluded from participation. Previous studies found that single-parenting [33], mental health disorders [32, 33, 59] and low socioeconomic status [60, 61] may negatively impact parental HRQL. The composition of our study sample may therefore limit the generalizability of our findings.

Large parts of this study were unexpectedly conducted during the COVID-19 pandemic, with all parents recruited after March 2020 being affected by visitation restrictions. Previous studies examining pandemic impact on parents in the NICU found that parents struggled with limited access to their infants and the inability to visit together with their spouses [62–64]. Parents reported that they felt visitation restrictions had increased their NICU-related stress [65]. Therefore, our observations may not be as applicable to experiences of parents outside of a pandemic setting.

Knowledge of gender-specific HRQL among parents of infants in the NICU remains sparse, and additional research is needed to assess the replicability of the results of this study and the variables that influence maternal and paternal HRQL. Gaining insight into the factors that influence parental HRQL at different points throughout childhood may be helpful in targeting interventions to better support affected parents.

## Data Availability

Participant-level data will not be disclosed to third parties for data protection reasons. The final trial dataset is accessed only by the principal investigator and research team members directly involved in the study. Study-related materials will either be part of the publication or will be available upon reasonable request.

## Abbreviations

CI: Confidence interval
GA: Gestational age
HRQL: Health-related quality of life
NICU: Neonatal intensive care unit
PedsQL™ FIM: Pediatric Quality of Life Inventory Family Impact Module
IQR: Interquartile range
PSOC: Parenting Sense of Competence Scale
SD: Standard deviation
VLBW: Very low birthweight
